# A New Method of Forming Clasps for Teeth

**Published:** 1859-04

**Authors:** T. D. Thompson

**Affiliations:** Providence, R. I.


### ARTICLE VII.

### *A new Method of Forming Clasps for Teeth.* By T. D. THOMPSON, D. D. S., Providence, R. I.

IF the subject of mechanical dentistry has not become obsolete as a subject upon which a thought may be publicly expressed, we would like to present the readers of the Journal a few observations on that part of dental art.

We are well aware that the members of the dental profession are quite notional; we all have our hobbies; what profession or calling has not?

With some of our worthy practitioners sponge gold is *the* gold; others as highly prize the adhesive; and others, still, are quite satisfied with Abbey's new adhesive foil. In these particulars we very much resemble Jonny Spratt and his very worthy spouse.

We possibly may find the same differences of opinion existing in regard to our present theme, namely, clasping teeth.

Some practitioners favor broad—others, narrow clasps, and we presume, in this age of advanced skill, many discard them as useless. Certainly it would be desirable could they be dispensed with.

The dentist of the broad clasp school would say that narrow clasps are more injurious to the teeth than are the broad, as in various ways they are liable to wear into the substance of the tooth: for instance, as in the motion of the plate often in masticating, or when the clasp does not grasp the tooth firmly, allowing the plate to cant, thus bringing the edges of the clasp in contact with the tooth in such a manner as to wear its substance; whereas, no canting or motion of the plate can cause a broad clasp to fret the tooth substance.

From a practical experience of several years, we are convinced that broad are less objectionable than are the narrow clasps. We will not insist, however, that any follow our notions, for as in the foil question, each party can adopt his own preferences.

Discarding wandering, we will present the reader with our method for forming a perfectly fitting clasp, broad or narrow. We in the first place secure an accurate form of the tooth to be clasped; next proceed to make a pattern of the clasp desired; we next cut from a piece of platina (rolled quite thin) a clasp; we burnish this clasp to the form of the tooth, carefully removing the same from the model; we place upon a piece of coal, placing through the imaginary centre, a tack; we fill the enclosed space with sand and plaster; borax well the surface; cut scraps of gold plate as we do solder, place them upon the clasp frame, and with the blow-pipe run them over the frame—build up the clasp to the thickness desired. By this method a perfectly fitting clasp is formed, which will need no bending to adjust it. We have formed our clasps as described for three or four years, and we conceive that a tooth cannot be injured when a clasp is thus formed—the adaptation being so perfect as to exclude all foreign matter from penetrating between the tooth and the clasp; it is in fact almost impervious to the fluids of the mouth.

